# Qualitative analysis of Adenomatous Polyposis Coli promoter: Hypermethylation, engagement and effects on survival of patients with esophageal cancer in a high risk region of the world, a potential molecular marker

**DOI:** 10.1186/1471-2407-9-24

**Published:** 2009-01-17

**Authors:** Maryam Zare, Ferdous Rastgar Jazii, Mohammad Reza Alivand, Negin Karimi Nasseri, Reza Malekzadeh, Mansour Yazdanbod

**Affiliations:** 1 Department of Biochemistry, National Institute of Genetic Engineering & Biotechnology (NIGEB), Tehran, Iran; 2 Division of Cell and Molecular Biology, Khatam University, Tehran, Iran; 3 Donnelly Center for Cellular and Biomolecular Research (CCBR), University of Toronto, Toronto, Canada; 4 Digestive Diseases Research Center, Shariati Hospital, Medical University of Tehran, Tehran, Iran; 5 Department of Surgery Shariati Hospital, Medical University of Tehran, Tehran, Iran

## Abstract

**Background:**

Squamous cell carcinoma of esophagus (SCCE) occurs at a high incidence rate in certain parts of the world. This feature necessitates that different aspects of the disease and in particular genetic characteristics be investigated in such regions. In addition, such investigations might lead to achievement of molecular markers helpful for early detection, successful treatment and follow up of the disease. Adenomatous Polyposis Coli (*APC*) promoter hypermethylation has been shown to be a suitable marker for both serum and solid tumors of adenocarcinoma of esophagus. We investigated the status of *APC *promoter hypermethylation in Iranian patients, compared the results with the former studies, and evaluated its applicability as a candidate molecular marker by examining association between survival of SCCE patients and *APC *promoter methylation.

**Methods:**

For evaluating the status of *APC *promoter hypermethylation and its association with SCCE, a qualitative methylation specific PCR (MSP) was used. DNA was extracted and digested with an appropriate restriction enzyme, treated with sodium bisulfite in agarose beads and amplified in two-step PCR reaction by applying either methylated or unmethylated promoter specific primers. Universally methylated DNA and methylase treated blood DNA of healthy donors were used as positive controls as well. Survival of patients was followed up for two years after treatment and survival rate of patients with methylated *APC *promoter was compared with that of unmethylated patients.

**Results:**

Assessment of *APC *promoter methylation revealed that normal tissues were unmethylated, while twenty out of forty five (44.4%) tumor tissues were hypermethylated either in one or both alleles of *APC*. Among the tissues in which methylation was detected, seven were hypermethylated in both alleles while the other thirteen were hypermethylated in one of the two alleles of *APC*. Analyzing two-year survival rate of patients with respect to promoter hypermethylation showed a lower rate of survival for patients with methylated *APC *promoter following their treatment. Further investigation into the association between promoter hypermethylation and tumor differentiation status indicated that patients with well differentiated tumors were more likely to develop promoter hypermethylation.

**Conclusion:**

Observing similar level of *APC *promoter hypermethylation in patients with SCCE in this high risk region and comparing it with other parts of the world could support the hypothesis that a common molecular mechanism might be involved in tumorigenesis of SCCE. In addition, the higher rate of two-year survival for patients with unmethylated *APC *promoter as well as its relationship with tumor differentiation would suggest that this tumor suppressor could be an appropriate candidate molecular marker for evaluating tumor malignancy and predicting survival of patients subsequent to treatment.

## Background

A high incidence rate of SCCE has been reported for the so-called Asian esophageal cancer belt; the highest rate of which was reported from Iran [1-5]. In addition, recent reports have evidenced an increasing incidence of esophageal cancer in developed countries, especially adenocarcinoma of esophagus [6-8]. Several genetic and epigenetic alterations have been suggested to play an important role in the carcinogenesis of esophageal and other gastrointestinal (GI) tumors; affecting different oncogenes, tumor suppressor genes, apoptosis regulating genes or mismatch repair genes such as *APC, P53, P16, DCC, RB, MCC, BRCA *and *MTS1/CDK41 *[9-18]. Hypermethylation of CpG islands in promoter regions of genes is a common epigenetic event of gene silencing in both types of esophageal cancers and impacts a wide range of important genes such as those involved in matrix remodeling like *TIMP3 *[19,20], ligand dependent suppressor genes for instance *DCC *[21], cell adhesion genes including cadherins (*CDH1*) and integrins [22-24], cell cycle regulator genes such as *p14*, *p16 *[20,23,24], apoptosis associated genes like *DAPK*, DNA repair and mismatch repair genes such as *MGM, hMLH1 *[20,23-25], xenobiotic metabolism engaged genes for instance *GSTP1 *[24] and *nel-like1*gene [26]. Epigenetic silencing of tumor suppressor genes has been shown to be associated with tumor invasiveness, growth, neovascularization, metastatic behavior and in particular, might be the cause of tumor recurrence after treatment, impacting overall patient survival [27-30].

Among those genes which are subject of epigenetic regulation, APC promoter hypermethylation occurs in all organs of GI cancers, both in hereditary and sporadic syndromes. This might indicate the importance of *APC *inactivation in tumorigenesis of these organs [31]. Moreover, recent reports indicate that *APC *is an important prognostic indicator for unfavorable clinicopathological outcome and tumor recurrence in several types of cancers [29,30]. *APC *along with several other hypermethylated genes play a prognostic indicatory role in squamous cell [22] and adenocarcinoma of esophagus [26], bladder [30], and lung cancers [29]. In fact, in adenocarcinoma of esophagus, *APC *promoter hypermethylation has been observed in 92% of cases [32]. This indicates that inactivation of *APC *plays a key role in the carcinogenesis of esophageal cancers and could be considered as a candidate molecular marker.

The Adenomatous Polyposis Coli (*APC*) tumor suppressor gene, maps on chromosome 5q21-22, has been investigated in several types of cancers and in particular colorectal cancers. While loss of heterozygosity [11,18,33,34] along with mutational inactivation [35,36] has been suggested for *APC *in esophageal cancer, nevertheless, mutations in *APC *are rare in this cancer [37-39]. Investigations have shown that inactivation of *APC *leads to increased β-catenin transcriptional activity and subsequent loss of cellular growth control. In normal cells, free β-catenin anchors to APC tripartite complex, composed of Axin-APC-GSK3-β and undergoes phosphorylation by glycogen synthase kinase3-β (GSK3-β), followed by proteasomal degradation, which results in reduction of free β-catenin in cytoplasm and thus, leading to silencing of the Wnt signaling target genes [40]. In contrast, loss of APC results in nuclear accumulation of β-catenin, which subsequently binds to Tcf-Lef (T cell factor/lymphoid enhancer factor) family of transcription factors, culminating in the activation of transcription and ultimately uncontrolled cell growth [41,42].

With regards to epigenetic regulation of *APC *and other tumor suppressor genes, so far as we know there is no previous report from this high risk region of the world. This is the first effort that has focused on epigenetic regulation of *APC *as an example of tumor suppressor genes such as *P15*^*INKb*^. We have recently begun to investigate their possible roles in carcinogenesis of SCCE in this region. The present report is the first attempt toward applying qualitative methylation specific PCR to reveal the methylation status of *APC *promoter within an Asian population highly at risk for developing SCCE. Our results show that the frequency of *APC *promoter methylation is almost the same as other regions, in particular the Western world. The results of this study also indicate that *APC *promoter methylation could be considered as a potential molecular marker for follow up the progress and survival of patients in consequence to their treatment.

## Methods

### Patients and specimens

45 patients including 27 men ranging in age from 23 to 80 (average of 61.1) and 18 women ranging in age from 45 to73 (average of 61.7) at the time of diagnosis were included in this study. Tumor tissues along with their adjacent normal tissues were used for analysis. Tissue samples were obtained from patients whom had not received chemo or radiotherapy before operation. Following surgery, tissue samples were snap frozen in liquid nitrogen and kept at -70°C until the time of DNA extraction. Tissues were included 32 poorly differentiated, 5 moderately and 8 well differentiated tumors according to the World Health Organization criteria (WHO, 1977). All samples were diagnosed as SCCE and status of differentiation were confirmed by pathological examination. Peripheral blood samples of healthy donors were collected as negative control. The same blood samples were also used for further treatment with DNA methylase for evaluating bisulfite treatment procedure. The survival rate of patients was monitored for two years. All patients and healthy donors of blood samples gave consent according to institutional guidelines and the study was approved by the research ethics committee of Digestive Disease Research Center (DDRC) of Medical University of Tehran.

### DNA Isolation and digestion

Genomic DNA was extracted from the ground frozen normal and tumor tissues following digestion with proteinase K and phenol/chloroform protein precipitation [43,44]. Blood DNA was extracted as previously described [45]. Subsequently, extracted DNA of either tumor or normal tissues was digested with Hind III (Fermentase Company), for which no restriction site is present in the entire 1556 bp of *APC *promoter. Digestion with HindIII enhances DNA denaturation and results in better bisulfite treatment as well.

### Agarose/DNA beads preparation

One μg of the digested genomic DNA was boiled for 5 min, immediately chilled on ice, and subsequently incubated in 0.3 M NaOH for 15 min at 50°C. Two volumes of the melted 2% low melting point (LMP) agarose (Roche Company) dissolved in ddH2O were pipetted into the mixture. DNA/agarose mixtures containing 100–200 ng of DNA were injected into chilled mineral oil to form agarose beads [46].

### Sodium bisulfite treatment of agarose beads

Deamination of DNA was performed using freshly made bisulfite solution (2.5 M sodium metabisulfite and 125 mM hydroquinone, pH 5) at 50°C in the dark. Bisulfite treatment of agarose beads at moderate temperature (50°C), for short time, reduces DNA degradation while still keeping it in the single stranded conformation required for complete treatment. Furthermore, since treated agarose beads could directly be used in PCR reaction, there is no need for precipitation of treated DNA which usually accompanied by some loss [46-48]. Aliquots of 200 μl bisulfite solution were added to vials containing a single bead and incubated at 50°C for 4 h in the dark [32,46]. Further treatment was stopped by equilibrating the beads with TE buffer (10 mM Tris HCL, 1 mM EDTA, pH 8) for 6 times, each time for 15 min, followed by desulfonation with 0.2 M NaOH, and neutralization with 1/5 (V/V) 1 M HCl for 15 min, this procedure was repeated twice. Finally, each bead containing modified DNA was washed twice with TE buffer and then twice with ddH2O, consecutively, each time for 15 min [46]. Beads were kept in a small volume of TE (pH 8) at 4°C and used in less than 3 weeks without any effect on the quality of MSP.

### Methylation Specific PCR

Bisulfite treated DNA from either of following samples was used in methylation specific PCR [49]. Samples were composed of normal and tumor tissues along with blood of healthy donors as negative control. In addition, methylated DNA (CpGenome Universal methylated DNA, Chemicon) and blood DNA modified by CpG methylase (New England Biolabs) were used as positive control and control of bisulfite treatment efficiency.

Methylation specific PCR was carried out using promoter 1A of *APC *in two-step amplification procedure. A primary amplification was followed by secondary methylation specific PCR. Bisulfite treatment was performed before primary amplification because DNA polymerase uses deoxycytosine in the reaction mixture wherever a guanine is present in the template. As a result, it becomes impossible to discriminate methylated from unmethylated cytosines if bisulfite treatment is done after primary amplification. Moreover, treatment after primary amplification also results in loss or degradation of DNA. For primary amplification, the region of promoter 1A without CpG dinucleotides was used for forward and reverse primer design. This approach not only verifies proper treatment of DNA but also provides an adequate template for the second round of PCR, in which methylated cytosines are differentiated from unmethylated ones by applying methylated CpG dinucleotides specific primers. Two sets of primers were designed for primary amplification. The first set of primers were the forward primer; 5'-TTT GTT TGT TGG GGA TTG GGG T-3', and the reverse primer; 5'-AAA CCC TAT ACC AAA AAA AAA CCA TC-3', resulting in a product of 402 bp. The second set of primers for primary amplification were the forward primer; 5'-GTT AGG GTT AGG TAG GTT GTG-3', and the reverse primer; 5'-AAA ACA ATA CAA AAA AAA ACC ACC TTC-3', leading to a 320 bp product. Bisulfite treated DNA was amplified in a 50 μl reaction volume containing 1× reaction buffer, 0.2 mM each dNTP, 1 mM MgCl2, 8 mM β-mercaptoethanol (2-ME), 0.8 μg/ml bovine serum albumin (BSA), 8% dimethyl sulphoxide (DMSO) and 20 pmol/reaction of either sets of primers designed for primary amplification. Applying a mixture of several PCR enhancers (DMSO, 2ME, BSA) increases the yield of MSP and the specificity of PCR products [50-52]. This is especially important in the case of GC-rich targets as well as for regions capable of forming secondary structure, which often result in little or no amplification. Cycling condition was composed of hot start at 94°C for 5 min before addition of 1.2 units of Taq polymerase (Roche), 10 cycles for step I of amplification was composed of denaturation at 94°C for 50 sec, annealing at 62°C for 1 min (touch down 0.2°C/cycle), extension at 72°C for 1 min followed by 27 cycles of step II of amplification with denaturation for 50 sec at 94°C, 1 min annealing at 58°C, 1 min extension at 72°C followed by 10 min final extension at 72°C.

Primary amplification condition for the second set of primers was the same as above except for application of 2 mM MgCl2, and slight reduction in annealing temperature of steps I and II. Amplification was composed of 10 cycles of 50 sec primary denaturation at 94°C, 1 min annealing at 58°C (touch down 0.2°C/cycle) and 1 min extension at 72°C as step I of amplification, followed by 27 cycles of 50 sec denaturation at 94°C, 1 min annealing at 56°C, 1 min extension at 72°C and 10 min final extension at 72°C, as step II. Eventually, PCR products were run in 2% agarose, stained with ethidium bromide and observed under UV light.

PCR products of primary amplification were used for MSP. The primers for amplification of methylated cytosines of promoter 1A were the forward primer; 5' TAT TGC GGA GTG CGG GTC 3', and the reverse primer; 5' TCG ACG AAC TCC CGA CGA 3'. The unmethylated CpG dinucleotide specific primers were the forward primer; 5' GTG TTT TAT TGT GGA GTG TGG GTT 3', and the reverse primer; 5' AAC CAA TCA ACA AAC TCC CAA CAA 3'.

MSP was performed in 50 μl PCR reaction mixture containing 1× reaction buffer, 0.2 mM each dNTP, 6 mM MgCl2, 8 mM β-mercaptoethanol, 0.8 μg/ml BSA, 8% DMSO, 20 pmol/reaction mixture of methylated or unmethylated specific primers and 1.2 units of Taq polymerase. PCR condition was hot start denaturation at 94°C for 5 min, 10 cycles of 50 sec denaturation at 94°C, 40 sec annealing at 56°C (touch down 0.2°C/cycle), 40 sec extension at 72°C followed by 28 cycles of 50 sec denaturation at 94°C, 40 sec annealing at 54.5°C, 40 sec extension at 72°C and 10 min final extension at 72°C. Each set of primers (methylated or unmethylated specific primers) was used for amplification of tumor and normal tissue samples as well as negative and positive controls. PCR products were run in 2% agarose gel, stained with ethidium bromide and visualized by UV illumination.

### Statistical analysis

Fisher's exact test was used to examine the association between *APC *promoter hypermethylation and mortality rate in SCCE patients. Moreover, the Pearson Chi-Square test was performed to find out possible correlation. Statistical significance was defined as *P *< 0.05 for Fisher's exact test and *P *< 0.001 for Pearson Chi-Square test.

## Results

**Figure 1:**
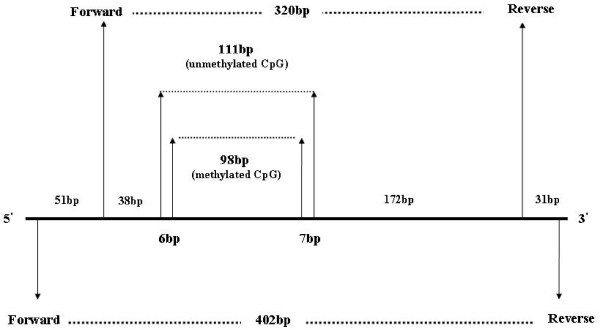
**The profile of *APC *promoter and the amplified products**. The products of primary amplification with primers designed for promoter sequence without CpG dinucleotides were either 402 or 320 bp. The product of the first step of PCR was used for the second step amplification by applying primers, which specifically distinguish methylated from unmethylated cytosines. The products of the second amplification were 98 bp for methylated and 111 bp for unmethylated cytosine in CpG islands of promoter.

**Figure 2:**
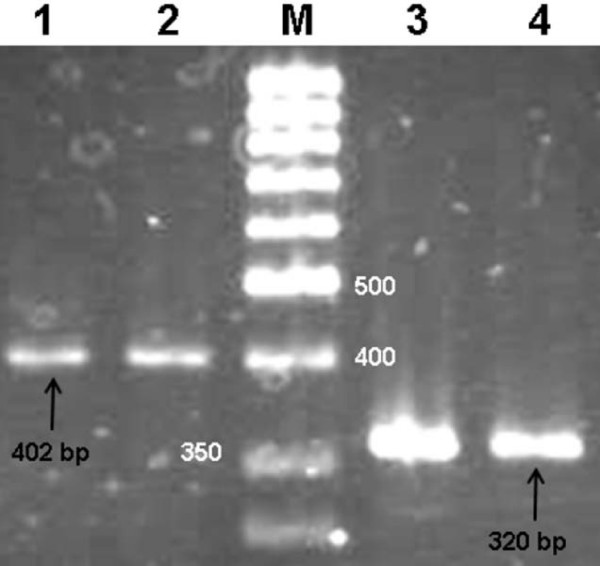
**PCR products of primary amplification subsequent to bisulfite treatment**. Lanes 1 and 2 show the 402 bp and lanes 3 and 4 the 320 bp PCR products. Either of the above PCR products could be used for secondary nested amplification.

To increase specificity of MSP procedure, four sets of primers were designed for promoter 1A of *APC *(NCBI accession No: U02509) (Figure 1). Two sets of primers were used in primary amplification leading to either 402 or 320 bp products, which were used in subsequent amplification by applying specific primers for either methylated or unmethylated *APC *promoter (Figures 1 and 2). The primers used in the primary amplification were designed against a part of the promoter sequence without CpG dinucleotides. This approach eliminated interference of methylated cytosines and the possible insufficiency of bisulfite treatment. In addition, enhanced discrimination of methylated from unmethylated cytosines became possible in subsequent MSP by applying primers specifically designed against part of the promoter with a high number of CpG dinucleotides (Figure 1). MSP products were either 98 bp for methylated cytosines or 111 bp in case of unmethylated.

**Figure 3:**
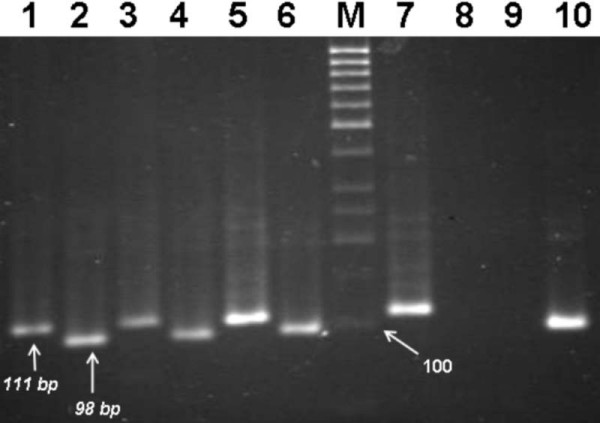
**MSP assessment of tumors versus normal tissues of three patients along with controls**. Lanes 1, 3 and 5 show unmethylated *APC *promoter amplification product of normal nonmalignant tissues with a 111 bp PCR product. Lanes 2, 4 and 6 are the corresponding methylated tumor tissues of *APC *promoter as could be observed with 98 bp PCR product. Lane 7; negative control (blood) which results in 111 bp product. Lane 10; positive control (universally methylated DNA) with a 98 bp product. Lane M; molecular size marker 50 bp (Fermentase).

**Figure 4:**
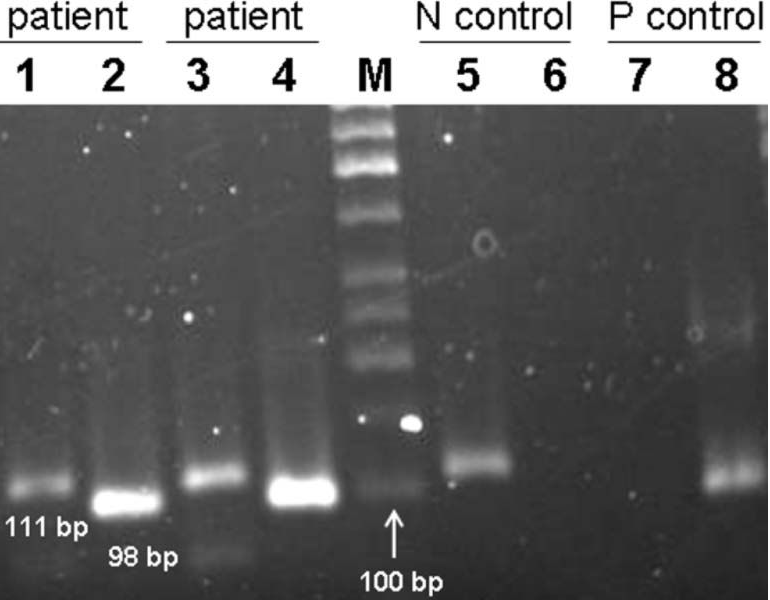
**MSP products of several hemimethylated or cellularly heterogeneous tumor tissues**. Both 98 bp MSP product of the methylated *APC *promoter and 111 bp MSP product of the unmethylated promoter could be observed in tumor tissues. Lanes 1 and 3 represent the MSP products of tumor tissues by applying unmethylated primers. Lanes 2 and 4 show the MSP products for the same tissues using methylated primers. Lanes 5 and 6; negative control (blood) which results in 111 bp product. Lanes 7 and 8; positive control (universally methylated DNA) with a 98 bp product. M; molecular size marker 50 bp (Fermentase).

Carrying out MSP on DNA extracted from normal tissues, 111 bp products were obtained, which indicate that normal tissues are unmethylated (Figure 3). In contrast, achievement of 98 bp amplification products for 20 out of 45 tumor tissues points out that 44.4% of tumor tissues are methylated either in one or both alleles of *APC*. Among methylated tumor tissues, 13 were heterozygous for the *APC *promoter; for which both 111 bp and 98 bp products were observed (Figure 4). This could happen due to two reasons; first, heterogeneity of tumor tissues regarding with their originated cells, such that both cells containing methylated and cells with unmethylated DNA are present in the same tissue. The second reason could be allelic heterozygosity of *APC *regarding with methylation. Conversely 7(16%) other tumor tissues were methylated for both alleles of *APC*, indicating that their corresponding tissues were homogeneous and composed of only one type of cells (Figure 3).

**Figure 5:**
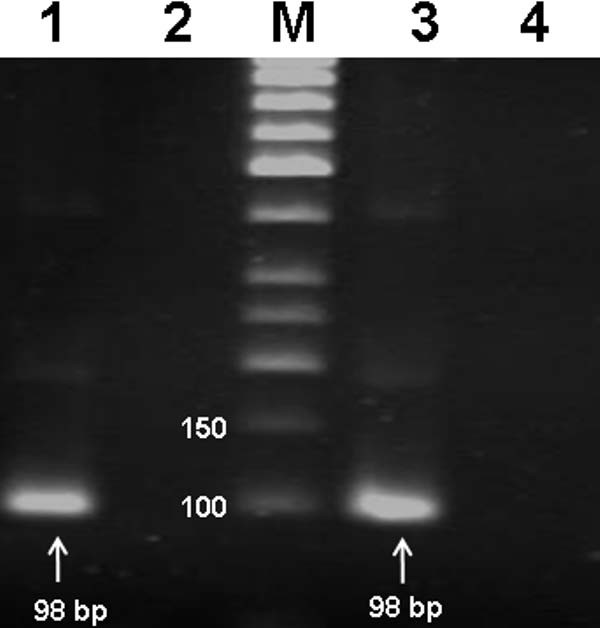
**MSP results of two positive controls**. Lane 1; MSP product of universally methylated DNA by applying methylated cytosine specific primers. Lane 2; the same as lane 1 but unmethylated cytosine specific primers were used. lane 3; MSP product of blood DNA extracted from a healthy donor treated with DNA methylase (CpG methyl transferase) and application of methylated cytosine specific primers, lane 4; the same as lane 3 but unmethylated cytosine specific primers were used.

To verify consistency of results, three controls were used; universally methylated DNA, blood DNA of healthy donors and DNA from the same donors treated with DNA methylase. As figure 5 shows following bisulfite treatment of blood DNA of healthy donors and carrying out MSP, it is negative for *APC *promoter methylation because it could only be amplified with unmethylated *APC *promoter specific primers. In contrast the same DNA could only be amplified by methylated *APC *promoter specific primers if it was treated with DNA methylase (Figure 5, lane 3). This provides evidence that bisulfite treatment was complete and MSP was properly carried out. Further support to our study was application of universally methylated DNA as positive control. Here amplification was only possible with methylated *APC *promoter specific primers. All the above controls verified that design of experiments was proper and the results are valid.

Pathological assessment of tissues showed that tumors were in different states of differentiation, such that 32 (71%) cases were poorly differentiated, 5 (11%) were moderately differentiated and 8 (18%) others were well differentiated. Among poorly differentiated tumors, 13 (41%) were methylated, while 19 (59%) were unmethylated. This condition was also true for moderately differentiated tissue samples in which 2 out of 5 (40%) tumors were methylated while 3(60%) others displayed unmethylated tumors. The highest rate of methylation was found in well differentiated tumors where 5 out of 8 (62.5%) tissues were methylated (Table 1).

**Table 1:** Methylation status and two-year survival of patients following surgery.

Tumor differentiation status *	Total	Survival rate	Methylated tumors	Survival rate	Unmethylated tumors	Survival rate
Poor	32	24	13 (40.6%)	8 (61%)	19 (59%)	16 (84%)
Moderate	5	4	2 (40%)	2 (40%)	3 (60%)	2 (67%)
well	8	4	5 (62%)	1 (20%)	3 (37.5%)	3 (100%)

Total	45	32	20 (44%)	11 (55%)	25 (56%)	21 (84%)

*Differentiation classification according to the WHO criteria (World Health Organization, 1977)

Following up survival of patients for two years revealed a clear relationship between patients survival and methylation status of *APC *promoter. Fisher's exact test, which examines the association between *APC *methylation circumstances and mortality of patients, showed the statistically significant correlation (P < 0.05). In addition, the Pearson Chi-Square test indicted the same relation. This statistic (X^2 ^= 6.86), at 2 degree of freedom (df), revealed that there is a significant association (p < 0:001) between presence of hypermethylated *APC *promoter and mortality rate among SCCE patients.

As Table 1 shows, patients with unmethylated tumors, in all states of differentiation, are more likely to survive for two or more years after treatment. Moreover, as differentiation status turns from well to poor, survival rate of patients with methylated promoters increases, while the converse is true for unmethylated promoters. It should also be noted that in the case of patients with moderate and well differentiated tumors additional samples are required to be included in future studies until a true judgment could be made.

## Discussion

Although epidemiological studies have indicated the highest incidence rate of SCCE occurs in Iran [1-5], nevertheless reports from this part of the world are limited. The present report is an extension to our former studies [4,10] on the molecular etiology of SCCE in this region, aiming to identify potential molecular markers. It is well known that tumor suppressor genes are mostly affected in SCCE [9,11,13-17,34,53-57]. As part of a long-term study we have started analysis of *APC *promoter methylation among tumor suppressor genes such as *p15*^*INKb *^(data not shown) as well as cell cycle inhibitors such as *p14*, *p15*, *p16 *and *p21*. Epigenetic regulation of gene expression through promoter methylation is one of the key means of controlling genes during development and also transcriptional silencing of tumor suppressor genes in cellular transformation. Promoter methylation pattern varies in different types of cancers. The highest occurrence of methylation has been observed in GI cancers involving both sporadic and inherited types [58].

*APC *is among tumor suppressor genes whose inactivation occurs in esophageal cancer as well as other GI cancers [31]. Inactivation of *APC *has been shown to be an early event in tumorigenesis of colorectal and gastric cancer [59-61], as could be observed with histopathological examinations and particularly in intestinal tumors in which sufficient levels of DNA methyltransferase activity play a role in the early polyp formation in *APC*^Min/+ ^mice [62]. Otherwise, *APC *hypermethylation has been observed in less advanced stages of both types of esophageal cancer, similar to *p16 *and *hMLH1 *genes [20,31]. Thus, *APC *could be considered as an appropriate predictive molecular marker especially for digestive tract cancers.

Eads and colleagues have previously shown *APC *promoter hypermethylation in Barrett's epithelium, either in metaplasia, dysplasia and adenocarcinoma of esophagus [54]. Meltzer's group [32] has demonstrated the significance of *APC *as a molecular marker for both serum and tissues of patients with adenocarcinoma of esophagus. Their study showed 92% hypermethylation of *APC *in adenocarcinoma. The same figure has also been obtained by Clement *et al. *[42], who have found *APC *promoter hypermethylation in all instances of Barrett's esophagus and in 95% of adenocacinoma of esophagus. Further study on the mucosa of patients at risk for developing Barrett's esophagus, a condition which progresses to adenocarcinoma of esophagus, has shown 88% methylation of *APC *promoter [63]. Moreover, in recent studies *APC *methylation has been found to be an appropriate molecular marker for monitoring tumor recurrence in lung [29]and bladder [30]cancer in which the presence of hypermethylated *APC *in the serum of patients correlates with worse clinicopathological features of malignancy.

These findings have encouraged us to study the status of *APC *promoter methylation in SCCE as well as evaluating its possible role as a potential molecular marker. Results indicate that 44.4% of patients with SCCE exhibited hypermethylation in the *APC *promoter. These patients were at a greater risk of death in the two years following treatment than the unmethylated patients. This finding indicates that examination of *APC *promoter could be applicable as a potential predictive survival marker for almost 50% of SCCE. In addition, combining this marker with other potential markers such as p53 for which a high frequency [10,16,33,64-66] of inactivation could be observed in SCCE would assist better treatment and follow up of disease. Achieving a 44.4% methylation of *APC *promoter might well point to the involvement of the same possible molecular alterations in the etiology of SCCE in the Iranian population as in other parts of the world. On the other hand, our observation for unmethylated normal esophagus epithelium is in agreement with Eads *et al. *[54], who have shown that normal esophageal epithelium is unmethylated for promoters such as *APC, CDH1, ESR1, CDKN2A*. Thus, it is rational to consider the methylation status of tumor suppressor genes' promoters as a potential marker for esophageal cancer.

Comparing our results with Meltzer's group [32] on SCCE reveals a close similarity (50% versus 44.4%), which might indicate *APC *to be among genes whose expression is affected at the same level in two distinct and geographically separate populations of the world. In addition, inactivation of *APC*, which results in β-catenin transcriptional activation [37,41,42,67], seems to be among prerequisites for esophageal carcinogenesis. Brabender *et al. *[28] have shown that high level of *APC *ptomoter hypermethylation is significantly associated with unfavorable clinical outcomes, lower survival rate and aggressive behavior of tumors. Our study also shows lower survival rate of patients with *APC *hypermethylation. The higher mortality rate of patients with methylated *APC *promoter indicates that *APC *is among determinant genes in esophageal carcinogenesis. Our former study on *p53 *tumor suppressor gene [10] further supports this notion as well as other former studies that have indicated the importance of tumor suppressor genes in the etiology of SCCE [9-18,33,34,36].

Our additional study on *p15*^INKb ^further shows the same pattern of promoter methylation in SCCE when, for example, our finding (16.6%) is compared with other reports such as Xing *et al. *[56] who have found 17.6% and Nie *et al. *[68] who have shown 19% of *P15*^*INKb *^hypermethylation. These results further indicate similarity in the process of tumorigenesis of SCCE.

There are two promoters for transcription of *APC*; promoter 1A and 1B [69]. In this study we focused on promoter 1A because this promoter is known to play a major role in carcinogenesis [70]. Previous studies in colon, breast, lung, endometrial and gastric cancers have indicated that promoter 1B is protected from methylation [70-72]. It should be noted that transcription might also start from promoter 1B; however, the product is an inactive protein. Nevertheless, further study on promoter 1B is recommended for a better understanding of the role of this promoter and its possible function in SCCE.

## Conclusion

Achieving a comparable pattern of *APC *promoter hypermethylation in the high risk region for SCCE, could be an indication for common molecular alterations in the etiology of SCCE between this region and other parts of the world. In addition, it raises hope for achieving a common molecular marker. Nevertheless, further studies are required to be carried out both on *APC *and other candidate genes, either at epigenetic level or at other molecular levels such as mutational inactivation and loss of heterozygosity. Identifying correlation between differentiation status and *APC *promoter methylation in conjunction with lower survival rate of patients with hypermethylated *APC *promoter implies the importance of epigenetic control of tumor suppressor genes in the tumorigenesis of SCCE, as well as the significant indicatory role of *APC *hypermetylation for evaluating tumor malignancy and predicting survival of SCCE patients subsequent to treatment.

## Competing interests

The authors declare that they have no competing interests.

## Authors' contributions

FRJ was the project leader and directed the study. He is the corresponding author. MZ was the main investigator of the study, performed the experiments and assisted in the draft of the manuscript. MRA and NKN participated in data acquisition and generated experimental data. RM is director of DDRC and supporter of the study as well as the head of the research ethics committee. MY was involved in surgery and tissue preparation. All authors reviewed and approved the final manuscript.

## Pre-publication history

The pre-publication history for this paper can be accessed here:

http://www.biomedcentral.com/1471-2407/9/24/prepub
